# Keratin 15, transcobalamin I and homeobox gene Hox-B13 expression in breast phyllodes tumors: novel markers in biological classification

**DOI:** 10.1186/1753-6561-6-S4-P21

**Published:** 2012-07-09

**Authors:** L Chong, PH Tan

**Affiliations:** 1 Royal College of Surgeons in Ireland, Ireland; 2 Singapore General Hospital, Singapore

## Introduction

Breast phyllodes tumors are rare neoplasms which present challenges for histological classification. Microscopic features are not always predictive of clinical behavior, and scarce data exist on the prognostic role of biological markers. Our study evaluated a series of 145 phyllodes tumors diagnosed at the Department of Pathology, Singapore General Hospital between 2006 and 2009, incorporating 91 (62.8%) benign, 40 (27.6%) borderline, and 14 (9.7%) malignant phyllodes tumors.

## Methods

Antibodies to keratin 15 (KRT15), transcobalamin I (TCN1), and homeobox gene Hox-B13 (HOXB13) were applied to sections cut from tissue microarray blocks. KRT15 and TCN1 positivity was defined when there was reactivity of 1% or more stromal cells, while HOXB13 positivity was defined using a H-score of 100 and above.

## Results

Positive immunohistochemical expression for KRT15, TCN1, and HOXB13 was seen in 21 (14.5%), 96 (66.2%), and 66 (45.5%) of tumors, respectively. Stromal expression of KRT15, TCN1, and HOXB13 was significantly correlated with tumor grade (P<0.001, P<0.001, P = 0.012), stromal hypercellularity (P = 0.005, P<0.001, P = 0.023), mitotic activity (P<0.001), and microscopic borders (P = 0.006, P<0.001, P = 0.011).

## Conclusions

Co-expression of TCN1 and HOXB13 was seen in 21 of 91 (23.1%) benign, 18 of 40 (45.0%) borderline, and 11 of 14 (78.6%) malignant tumors, suggesting that the dual-marker panels of TCN1 and HOXB13 might be helpful in classifying borderline and malignant tumors. Although expression of TCN1 alone was present in all malignant and 34 of 40 (85.0%) borderline tumors, a combined panel with HOXB13 excluded some benign cases and was a better discriminant for a significant proportion of borderline and malignant tumors.

